# Whole heart self-navigated 3D radial MRI for the creation of virtual 3D models in congenital heart disease

**DOI:** 10.1186/1532-429X-18-S1-P185

**Published:** 2016-01-27

**Authors:** Nicole Wake, Li Feng, Davide Piccini, Larry A Latson, Ralph S Mosca, Daniel K Sodickson, Puneet Bhatla

**Affiliations:** 1 grid.137628.90000000121698901Center for Advanced Imaging Innovation and Research (CAI2R) and Bernard and Irene Schwartz Center for Biomedical Imaging, Department of Radiology, New York University School of Medicine, New York, NY USA; 2 grid.137628.90000000121698901The Sackler Institute of Graduate Biomedical Sciences, New York University School of Medicine, New York, NY USA; 3 Advanced Clinical Imaging Technology, Siemens Healthcare, Lausanne, Switzerland; 4 grid.137628.90000000121698901Department of Cardiothoracic Surgery, New York University School of Medicine, New York, NY USA; 5 grid.137628.90000000121698901Department of Pediatrics, New York University School of Medicine, New York, NY USA

## Background

Three-dimensional (3D) virtual models are valuable tools that may help to better understand complex cardiovascular anatomy and facilitate surgical planning in patients with congenital heart disease (CHD). Although computed tomography (CT) images are used most commonly to create these models [1,2], Magnetic Resonance Imaging (MRI) may be an attractive alternative, since it offers superior soft-tissue characterization and flexible image contrast mechanisms, and avoids the use of ionizing radiation. However, segmentation on MRI images is inherently challenging due to noise/artifacts, magnetic field inhomogeneity, and relatively lower spatial resolution compared to CT. The purpose of this study was to evaluate the image quality and assess the feasibility of creating virtual 3D heart models using a novel prototype 3D whole heart self-navigated radial MRI technique.

## Methods

Free-breathing self-navigated whole heart MRI was performed on three pediatric patients: two with complex CHD (average age=17 months) and one with normal cardiac anatomy (age=17years), using a 3D radial, non-slice-selective, T_2_-prepared, fat-saturated bSSFP sequence on a 1.5T MRI scanner (MAGNETOM Aera, Siemens, Germany). The acquisition window (~50-55 ms) was placed in mid-diastole and was adapted for different heart rates. Imaging parameters were as follows: TR/TE=3.1/1.56 ms, FOV=200 mm^3^, voxel size=1 mm^3^, FA=115°, and acquisition time=5-6 minutes (~12000 radial lines). Respiratory motion correction and image reconstruction was performed on the scanner as described in [3]. For comparison, conventional non-gated 3D FLASH or navigator-gated 3D bSSFP sequences were also performed. All results were blinded and randomized for image quality assessment by one pediatric cardiologist and one cardiac radiologist using a five-point scale (1=non-diagnostic, 2=poor, 3=adequate, 4=good, 5=excellent). Statistical analysis was performed to compare mean scores. DICOM images were imported to a 3D workstation (Mimics, Materialise, Leuven, Belgium) for 3D post-processing. The cardiovascular anatomy was first segmented using a combination of automated and manual techniques; and volume rendering was performed to depict the anatomy of interest.

## Results

**Figure 1:**
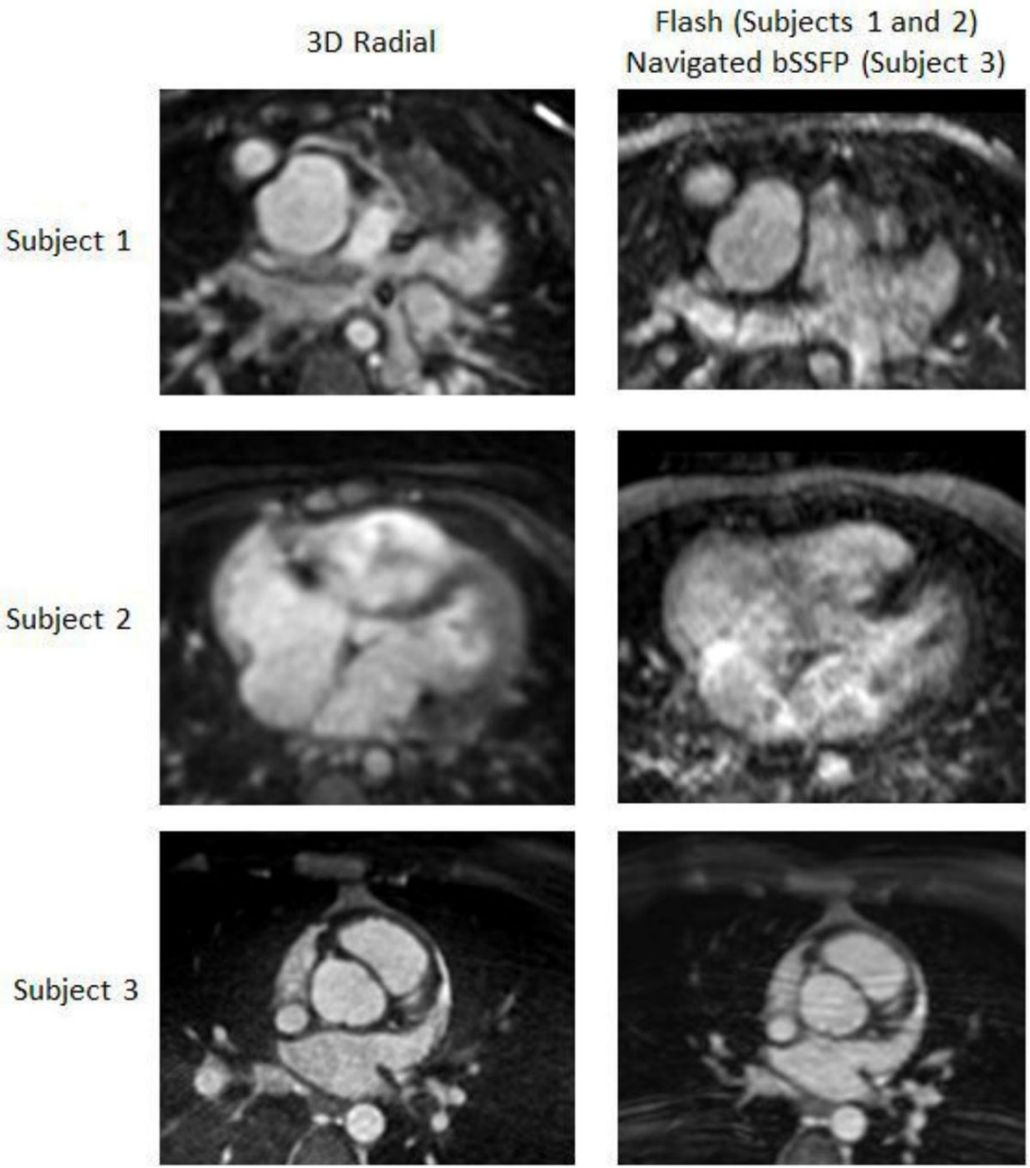
**Comparison between 3D Radial Sequence (left) to FLASH (Subjects 1 and 2) or respiratory-navigated bSSFP (Subject 3)**. Note the sharp myocardial wall contrast(Subjects 1 and 3) and tricuspid valve leaflets (Subject 2, Ebstein's anomaly) in the 3D radial sequence.

**Figure 2:**
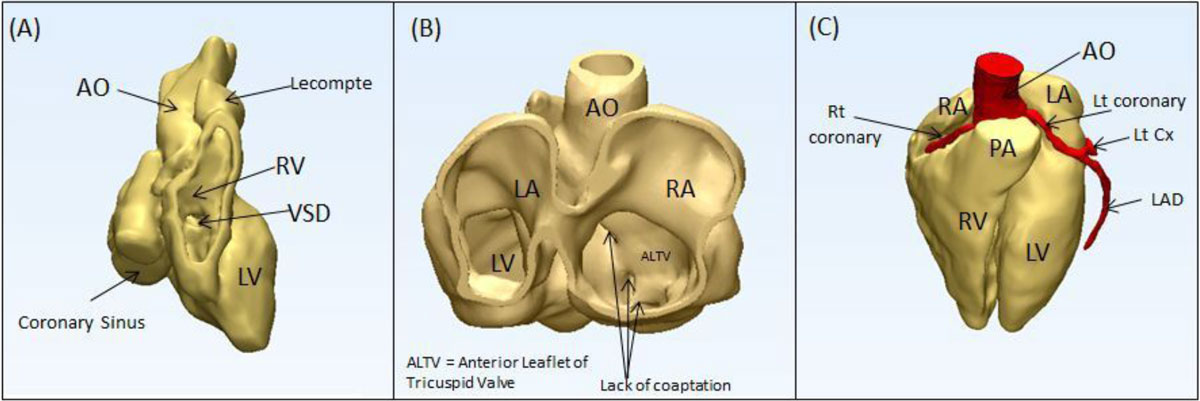
**3D Virtual models demonstrating (A) arterial switch with lecompte, VSD, and enlarged coronary sinus in subject 1, (B) area of tricuspid regurgitation due to lack of coaptation in subject 2 (Ebstein's anomaly), and (C) normal cardiac anatomy and coronary arteries in subject 3**.

The free-breathing self-navigated 3D radial acquisition provided significantly improved image quality and myocardial wall-blood contrast (Figure 1). Mean scores were 4.58 and 2.67 for the 3D radial and FLASH/bSSFP sequences respectively (p = 0.003). The cardiovascular anatomy was well depicted on all virtual 3D models (Figure 2).

## Conclusions

3D virtual models are frequently being created to understand complex anatomy, influence surgical planning, and provide intra-operative guidance for patients with CHD. This novel free-breathing, self-navigated whole heart 3D radial sequence provided excellent image quality as compared to existing routine MR sequences. Furthermore, the superb image quality provided using this novel sequence makes it an excellent choice for the creation of 3D models.

